# Vocal music and brain plasticity_a literature review

**DOI:** 10.1192/j.eurpsy.2023.559

**Published:** 2023-07-19

**Authors:** H. Arshad, G. Muhammad, A. R. Khan, A. Arshad

**Affiliations:** ^1^Psychiatry, Jinnah Sindh medical university; ^2^Medicine, Karachi Medical and Dental College, karachi, Pakistan; ^3^Psychiatry, Carilion Clinic Virginia Tech., Virginia, United States; ^4^Medicine, Jinnah Sindh Medical University, Karachi, Pakistan

## Abstract

**Introduction:**

Vocal music has been a way for the expression of beautiful human emotions and gives a consolidated framework to words. Our review is centered on finding neuroplastic changes in exposure to music.

**Objectives:**

Our main Objective is to identify structural brain changes in different brain areas. Identification of motor and sensory changes that are produced in response to vocal music.

**Methods:**

Detailed literature review was conducted using Pubmed and Google Scholar databases. The literature search was narrowed down to cover the research topic with the search terms [plasticity] OR [brain] OR [neurons] OR [music] OR [vocal]. Our Inclusion criteria included studies with effects of vocal music on neuronal plasticity regardless of age, gender, duration of training, type of training, medium of lanuage and profession. Exclusion criteria included instrumental music and forms of music other than vocal music.

**Results:**

Results showed that music impacts areas of the brain that are highly associated with human emotions. Any brain area can undergo neuroplasticity but is most commonly seen in the insular areas, paracortex, putamen, amygdala, and white matter. Music therapy promotes the formation of instant neural networks and the release of neurotransmitters like serotonin and dopamine. These microscopic changes increase depending on the duration of exposure to vocal music. Later, it appears as macroscopic changes visible with the help of neuroimaging. There is also a significant difference in the brain changes of vocalists and non vocalists. Vocal music impacts the left side of the cortex. Music activates reward system in the brain that leads to stimulation of dopaminergic pathways. It helps in neuronal division in post strock and post traumatic brain injury patients.

**Conclusions:**

Music therapy is widely used as the rehabilitative process that combines music with therapeutic medications to promote therapeutic alliance and better results. It is used to direct focus toward the fulfillment of the emotional and cognitive needs of patients with psychiatric ailments. This area is needed to be explored more so that vocal music can be used for integrated therapy.

Keywords: Vocal music; Brain changes; neuroplasticity; therapy.

**Disclosure of Interest:**

None Declared

